# Prediction and Analysis of Multi-Response Characteristics on Plasma Arc Cutting of Monel 400™ Alloy Using Mamdani-Fuzzy Logic System and Sensitivity Analysis

**DOI:** 10.3390/ma13163558

**Published:** 2020-08-12

**Authors:** Rajamani Devaraj, Emad Abouel Nasr, Balasubramanian Esakki, Ananthakumar Kasi, Hussein Mohamed

**Affiliations:** 1 Centre for Autonomous System Research, Department of Mechanical Engineering, Vel Tech Rangarajan Dr. Sagunthala R&D Institute of Science and Technology, Chennai 600062, India; balasubramaniane@veltech.edu.in; 2 Department of Industrial Engineering, College of Engineering, King Saud University, Riyadh 11421, Saudi Arabia; eabdelghany@ksu.edu.sa; 3 Department of Mechanical Engineering, Faculty of Engineering, Helwan University, Cairo 11732, Egypt; hussein@h-eng.helwan.edu.eg; 4 Department of Mechanical Engineering, Karpagam College of Engineering, Coimbatore 641032, India; ananthakumar.k@kce.ac.in

**Keywords:** ANOVA, kerf taper, heat affected zone, material removal rate, response surface methodology, intelligent modeling

## Abstract

Nickel-based alloys, especially Monel 400™, is gaining its significance in diverse applications owing to its superior mechanical properties and high corrosion resistance. Machining of these materials is extremely difficult through the traditional manufacturing process because of their affinity to rapid work hardening and deprived thermal conductivity. Owing to these difficulties a well-established disruptive metal cutting process namely plasma arc cutting (PAC) can be widely used to cut the sheet metals with intricate profiles. The present work focuses on an intelligent modeling of the PAC process and investigation on the multi-quality characteristics of PAC parameters using the fuzzy logic approach. The Box-Behnken response surface methodology is incorporated to design and conduct the experiments, and to establish the relationship between PAC parameters such as cutting speed, gas pressure, arc current, and stand-off distance and responses which include the material removal rate (MRR), kerf taper (KT), and heat affected zone (HAZ). The quadratic regression models are developed and their performances are assessed using the analysis of variance (ANOVA). Fuzzy set theory-based models are formulated to predict various responses using the Mamdani approach. Fuzzy logic and regression results are compared with the experimental data. A comparative evaluation predicted an average error of 0.04% for MRR, 0.48% for KT, and 0.46% for HAZ, respectively. The effect of variations in PAC process parameters on selected responses are estimated through performing the sensitivity analysis.

## 1. Introduction

Cutting-edge engineering materials including composites, superalloys, and ceramics are revolutionizing the industrial demand owing to their superior material properties such as high strength-to-weight ratio, increased corrosive and wear resistance, and are also able to withstand at elevated temperatures [1]. Alloys in general, nickel and titanium in particular, are mostly utilized for high temperature applications to obtain minimal deformation and high thermal stability [2]. Exclusively, Monel 400 which is a nickel-based alloy is being widely used in marine ship building, aircraft engines, nuclear and chemical processing industries [3]. Owing to its enhanced mechanical properties such as inferior thermal conductivity, high toughness, better creep resistance, and superior yield strength, as well as ease in cutting of intricate part profiles, which is extremely difficult to cut using traditional cutting processes, made Monel 400 a potentially viable material in today’s manufacturing sectors. In addition, advanced metal cutting processes such as laser cutting, abrasive waterjet cutting, electrical discharge machining, plasma arc cutting, ultrasonic machining, etc. are very well suited for processing these kinds of difficult-to-cut materials [4,5,6,7].

Among these non-traditional manufacturing processes, the plasma arc cutting (PAC) technology is a well-recognized thermal energy based on the non-traditional machining process extensively practiced for processing of widespread materials especially all electrically conductive materials and superalloys [8]. Compared with a range of available traditional and non-traditional machining processes, PAC exhibits a high potential due to its excellent speed of cutting, low cost, and manifest mechanization. In the PAC process, a high intensity constricted plasma arc is produced between the electrode and workpiece material by the ionization of gas through the application of high intensity arc current. The high temperature arc of above 20,000 °C is used to melt the material and subsequently the melted metal is ejected from the cutting zone by the application of high-pressurized gas supply. At this junction, plasma is generated inside the torch and the ionization of cutting gas leads to the plasma state. A high transfer of heat energy into the workpiece causes melting and the ejection of smelted metal is achieved through kerf. PAC is considered to be cost efficient in comparison to other machining processes to fabricate complicated profiles, it is able to machine the parts of a wide range of materials effortlessly and rapid cutting is regarded as the most versatile non-conventional machining process. Even though PAC has more potential benefits, achieving high quality of cutting with reference to HAZ, surface roughness, and kerf characteristics is deliberated to be challenging because the influence of diverse PAC process parameters affects the performance. It is essential to investigate the key contributing PAC process variables and examine the influence in terms of part quality characteristics. This study also signifies and apprehends the meagerness of the PAC process in meeting the demand of manufacturing industries.

Most of the earlier works concentrated on theoretical and experimental strategies to evaluate the part quality and machining characteristics such as HAZ, MRR, recast layer formation, surface roughness, and geometrical kerf qualities. In the literature, few PAC studies are performed for machining metals, ceramics, and composites. Adalarasan et al. [8] investigated the consequence of various process parameters such as arc current, torch stand-off, cutting speed, and gas pressure on quality characteristics of the PAC process such as surface roughness and kerf taper during cutting of a 304 L stainless steel using the grey taguchi statistical hybrid approach. They found that the quality of plasma arc cutting parts can be enhanced through maintaining lower arc current and stand-off distance. Ramakrishnan et al. [9] utilized genetic algorithm for optimizing the PAC parameters to obtain enhanced cut quality characteristics during cutting of a SS321 steel. Their results reveal that the enhanced surface quality and minimized heat affected zone can be obtained by reducing the cutting speed. Further, they have reported that metaheuristic optimization techniques such as genetic algorithm can be effectively used for optimization of the PAC process. Salonitis et al. [10] experimentally investigated the heat affected zone, cut quality, and conicity of cut geometry of a PAC processed S235 mild steel. They have proposed that the HAZ can be reduced by minimizing the arc current and the stand-off distance which are the most influencing factors on the cut quality of conicity of cut geometry. Celik [11] studied the effects of cutting parameters such as cutting speed, arc current, and arc voltage on the plasma arc cutting of sheet metals with different thickness. His investigation results proposed that the heat affected zone increases when the cutting speed decreases, whereas surface roughness decreases with the reduction of the cutting speed. Abdulnasser et al. [12] experimentally investigated the rate of material removal and surface quality of an aluminium sheet with different thickness. Their results indicated that the cutting characteristics are mostly influenced by the arc current and cutting speed followed by the stand-off distance. Subbarao et al. [13] investigated the PAC performance characteristics during cutting of a Hardox–400 alloy by varying its governing parameters using the design of experiments. They have found that the irregularity in cutting surface has been controlled by decreasing the cutting speed and arc voltage. Maity et al. [14] studied the impact of PAC parameters on the cut quality for an AISI 316 stainless steel. The results show that the cut quality is solely influenced by the stand-off distance whereas other parameters have no obvious impact. Gariboldi et al. [15] investigated the quality responses of PAC on a titanium sheet with varying thickness and found that the oxygen and nitrogen shield gases that were produced enhanced the cut quality.

Earlier studies of the authors dealt with various optimization techniques to investigate the PAC process. Rajamani et al. [16] investigated the influence of PAC parameters on kerf width, surface roughness, and microhardness of the machined Monel 400 alloy. They have proposed a statistical desirability approach for the optimization of PAC process parameters. Results of their investigation reveal that the surface roughness is significantly influenced by the stand-off distance and arc current, whereas kerf width and microhardness are influenced by the cutting speed and gas pressure. In another study [17], they have successfully utilized the TOPSIS statistical approach for the optimization of PAC process parameters to improve the cut qualities such as material removal rate, minimized kerf taper, and heat affected zone.

These studies are utilized as conventional methods of modeling and optimization which necessitate a lot of experimentation that is time consuming and huge efforts are required to achieve the desired part quality. In order to alleviate from these issues, various artificial intelligent (AI) techniques are employed in the literature to model the system and evaluate the performance of the developed system. However, fuzzy logic (FL) is considered to be a prominent AI technique, comprising of linguistic terms with reference to establishing a membership function for the given input and output parameters that has more degree of uncertainty and vagueness [18]. Therefore, FL is fascinating in its attention to solve complex mathematical and engineering problems in various fields. In recent times, many of the researchers have implemented the FL theory for modeling of advanced manufacturing processes.

Rahul et al. [19] exploited the performance characteristics of electric discharge machining on the Inconel superalloy using a hybrid fuzzy-Taguchi approach. Kuriachen et al. [20] utilized FL and particle swarm optimization for the development of the system and optimization of the micro-wire electric discharge machining process. They reported that a high correlation exists between fuzzy predicted system and experimental values that have proven that the developed system is accurate and demanding. Hossain et al. [21] experimentally studied the kerf quality of laser beam machining using the fuzzy expert system. The findings of their research show that the Mamdani fuzzy approach can be used for investigating the kerf quality of the laser machining process with a lower relative error and higher prediction efficiency. Bikash et al. [22] incorporated the grey-fuzzy methodology to investigate the influence of wire electric discharge machining (WEDM) variables on quality characteristics. The validation results of their investigation designate that the suggested approach can be successfully used for modeling and optimization on WEDM of AISI steels.

Later on, Prabhu et al. [23] presented the fuzzy response surface methodology approach for building the system and performed optimization studies on the EDM process. They found that the developed fuzzy approach is utilized to map the affiliation among the machining variables and corresponding responses. Parthkumar Patel et al. [24] utilized the Mamdani based fuzzy logic intelligent approach to investigate the influence of PAC variables on the surface quality of metal removal rate during cutting of an AISI D2 steel. Cebeli et al. [25] studied the surface quality of an AISI 4140 steel during plasma arc cutting by varying the process parameters. They found that the fuzzy approach can be effectively used for analyzing the impact of input parameters on quality responses.

Intelligent modeling of the PAC process leads towards the continual improvement of quality characteristics in final products and processes including the modeling of input–output and in-process parameters through cost-effective modeling approaches such as FL, ANN, ANFIS, etc. Though the FL approach is widely used for modeling modern manufacturing processes, no obvious investigation is found to model the PAC process for machining of nickel-based alloy materials. Therefore, the present research work focuses on modeling of PAC using the FL technique and evaluating its performance characteristics. Firstly, experimental trials are planned and executed based on the Box-Behnken response surface method and a mathematical model is established. The competence of the established models is legalized using the multi-factor analysis of variance. Further, the Mamdani based fuzzy logic intelligent approach is exploited to associate the PAC process for specified input process parameters such as cutting speed, gas pressure, arc current, and stand-off distance in evaluating the output features such as MRR, KT, and HAZ. A comparative evaluation of the regression model, FL, and experimental results are performed. Finally, the quantitative influence of these PAC variables on selected responses is attained through conducting the sensitivity analysis.

## 2. Methodology

### 2.1. Response Surface Methodology

Response surface methodology (RSM) is employed for modeling and optimizing the governing parameters [26]. The Box-Behnken design (BBD) is a RSM-based approach which is utilized to minimize the experimental runs, establish the quadratic model, and also study the interactions between the PAC governing parameters. The second-order polynomial equation formed from RSM was utilized to manifest the behaviour of the PAC process as shown in Equation (1). In this investigation, the viscoelastic properties of sintered specimens are modeled in accounting the selected process parameters.

(1) $$Y=C_{0}+\sum_{i=1}^{n}C_{i}X_{n}+\sum_{i=1}^{n}d_{i}X_{i}{}^{2}\pm \varepsilon$$

In the present investigation, a second-order quadratic model is developed using RSM for correlating process variables and the responses. Additionally, ANOVA is exploited to justify the consequence of developed quadratic models.

### 2.2. Fuzzy Logic Expert System

**Figure 1:**
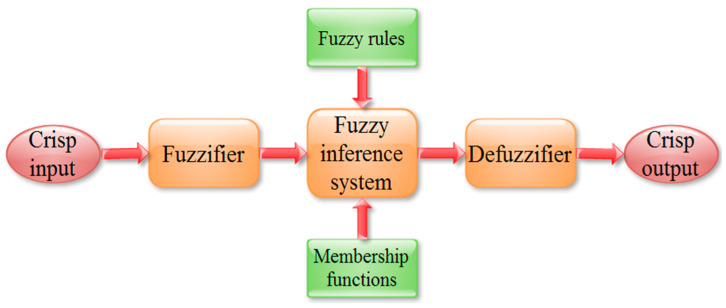
Schematic of the fuzzy inference system.

Fuzzy logic (FL) is proven to be an effective artificial intelligence (AI) technique for modeling the process parameters of complex processes which are largely probabilistic rather than deterministic [27]. FL consists of three conceptual components such as fuzzification (adapt crisp inputs into fuzzy values), fuzzy inference system (define fuzzy rules by adopting membership functions), and defuzzification (translate fuzzy outputs to crisp values) during the development of the model as shown in Figure 1. It is functioning based on imprecision; exclusively, it mimics the decision of human beings through uncertain and vogue information. Owing to its superior capability in establishing a relationship between input and output of any processes with a powerful decision-making ability bounded by minimal fuzzy linguistic rules, it is widely utilized in diverse applications in comparison to other statistical and AI techniques. The present investigation considers the FL approach to model the PAC process and is also useful to determine the appropriate PAC process parameters.

Generally, three categories of FL methodologies such as Mamdani, Tsukamoto, and Sugeno are employed to solve the non-linear problems [28]. However, due to the simplified structure, enhanced computational speed, and translating of rules, the Mamdani fuzzy inference engine is widely used to examine multifarious engineering issues. [29]. The present investigation considers input PAC process variables such as cutting speed (A), gas pressure (B), arc current (C), and stand-off distance (D) to evaluate the output responses namely MRR, KT, and HAZ. In FL, the construction of membership functions (MFs) are considered to be critical and it is the graphical illustration of extent of each process variable. Due to the existence of high computational efficiency and uncertainty during the splitting of values, triangular MFs are selected for modeling PAC process variables. The expressions in the MF curve to designate input process variables are selected as ‘Low’, ‘Medium’, and ‘High’ for input process variables. Similarly for output responses such as MRR, KT, and HAZ, the following expressions are accounted: ‘Very very low (VVL)’, ‘very low (VL)’, ‘low medium (LM)’, ‘low (L)’, ‘medium (M)’, ‘high (H)’, ‘high medium (HM)’, ‘very high (VH)’, and ‘very very high (VVH)’. The triangular MFs for input variables are specified using the following relation:

(2) $$Triangle\;\mathrm{MF}\;\left(a;x,y,z\right)=\left\{ \begin{array}{ll} 0, & a\leq a\\ \frac{a-x}{y-x}, & x\leq a\leq y\\ \frac{z-a}{z-y}, & y\leq a\leq z\\ 0, & z\leq a \end{array}\right.$$

where a is a variable, and x,y,z specifies fuzzy triangular levels. The MFs and Mamdani rules are intended to achieve preferred predictor variables. In this present study, thirty ‘IF-THEN’ fuzzy rules are developed to achieve the desired MRR, KT, and HAZ through utilizing MATLAB 8.5.0. Finally, the centroid method is utilized for defuzzification to extract the crisp output from the earlier formulated fuzzy set and the centroid is calculated using the following relation [30]:

(3) $$y_{o}=\frac{\sum y\times \left[\mu_{C_{i}}\left(y_{j}\right)\right]}{\sum \left[\mu_{C_{i}}\left(y_{j}\right)\right]}$$

The crisp value $y_{o}$ gives the output response value and $y_{j}$ indicates the center value of regions (i.e., responses).

## 3. Experimental Details

**Figure 2:**
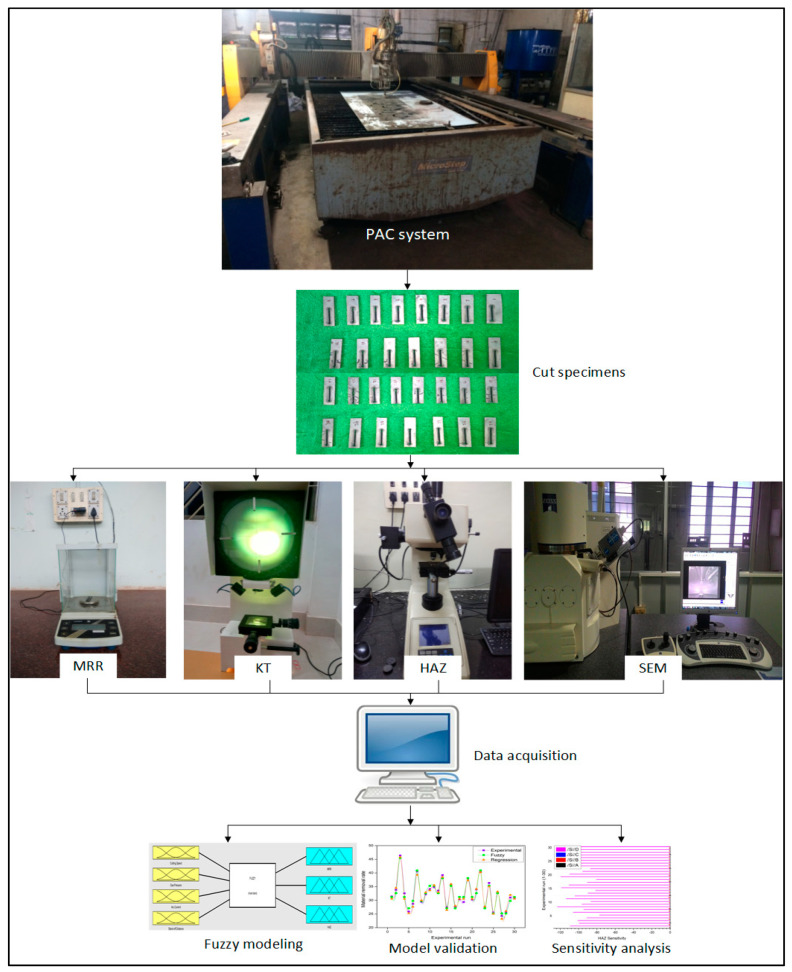
Methodology of the proposed plasma arc cutting (PAC) process.

Experimental investigations on PAC of the Monel 400 alloy having a material composition of 63% nickel, 31.6% copper, 2.5% steel, 2% manganese, 0.5% silicon, and 0.3% carbon is performed. A rectangular specimen of 3 mm thickness with 200 mm × 200 mm is considered as a workpiece material. Straight cuts of 25 mm long are made with two repetitions for each experimental run. The cut specimens are removed from the base material for recognizing the responses. The experimental investigations were performed using an industrial purpose plasma arc cutting system (Pro arc welding and cutting system, India). The PAC experimental system is presented in Figure 2. The PAC setup is furnished with the PlasmaCAM CNC software to confirm the accurate motion of plasma jet through the nozzle. Compressed air is used as a shield gas to generate high-energy plasma to thaw out and spew the smelted metal in the substrate surface. The precision in the cutting operation was accomplished through a servo-operated torch devising a copper nozzle with an air-cooled swirl.

A series of experiments are conducted according to the designed matrix and a range of selected process parameters as shown in Table 1. The range of selected PAC parameters are established with an aid of the existing literature, machine capability, and conducting exhaustive pilot experiments. A nozzle diameter of 1.5 mm, arc voltage of 110 V, and piercing time (0.3 s) are kept constant throughout the experiment. Each response is calculated based on experimental measures and tabulated for further investigations.

The removal of metal during the cutting process is calculated using the following expression:

(4) $$MRR\left(g/\min\right)=\frac{Metal\;removed\;from\;the\;workipece}{Cutting\;time}$$

**Figure 3:**
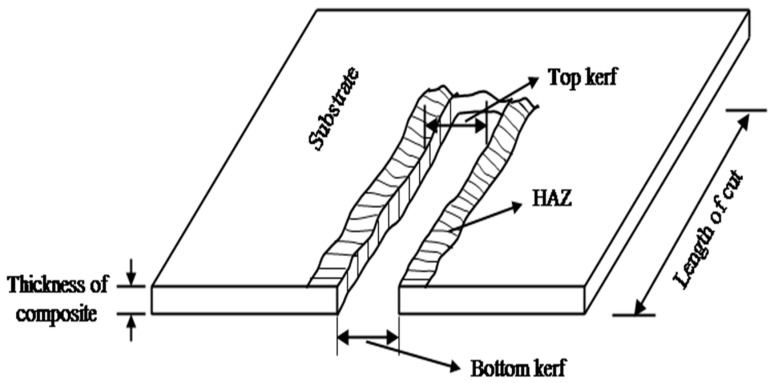
Kerf taper and heat affected zone measurements.

MRR is quantified using an Infra IN210 (Infra Instruments Ltd., Chennai, India) electronic weight balance with a precision of 0.0001 gms. Similarly, the kerf taper (Figure 3) is measured on an automated profile projector (Scientico Instruments, India) and the following relation is used to evaluate KT:

(5) $$Kerf\;taper\left(Degree\right)=\frac{\left(Top\;KW-Bottom\;KW\right)\times 180}{2\pi t}$$

where t is the thickness of the sheet and KW is the kerf width. For each sample, three measurements on the kerf width are conducted and their average are accounted.

The region of heat affected near the cut surface (distance perpendicular to the length of cut) is considered as HAZ and is measured using a tool makers microscope BX53 (Made: Olympus Corporation, Tokyo, Japan) with a magnification of 40×. The measured responses corresponding to various experimental trials are listed in Table 2. The surface morphology of the machined surface is assessed by a field-emitted scanning electron microscope (FESEM). The analysis is performed by using a Sigma 300 (Make: Carl Zeiss, Jena, Germany) apparatus with a maximum accelerating voltage of 15 keV.

## 4. Results and Discussion

### 4.1. Regression Modeling and Statistical Analysis

The second-order mathematical models are developed to statistically analyze the relative importance of PAC governing parameters. The competency of established mathematical models is substantiated through the analysis of variance (ANOVA) with a 95% confidence interval using the Design Expert 7 software. Table 3 shows the ANOVA outcomes for the quality characteristics such as MRR, KT, and HAZ. It is perceived from the tables that *p* < 0.05, which illustrates that the developed models are at a 95% assurance level. Moreover, the factor of determination (R2) for the MRR, KT, and HAZ are also found to be 0.9772, 0.9839, and 0.9904, respectively. It illustrates that the established mathematical models reasonably fit with the real data. The adequate precision (AP) values obtained for selected responses are well above 4, that signposts the acceptable model perception. The model F values denote that the models are significant. From the established models, certain variables can be observed as insignificant terms due to their “Prob. > F” value presence of more than 0.05. The backward elimination approach was utilized to remove the insignificant terms from the developed mathematical models. The remaining terms are retained for further estimation. The final mathematical models are given by:

(6) $$\begin{array}{l} MRR\left(g/\min\right)=894.842+0.0623\times A+3.757\times B-29.547\times C-137.812\times D\\ -0.01205\times A\times B+5.028\times 10^{-3}\times A\times C-4.327\times B\times D+2.041\times C\times D\\ -5.779\times 10^{-5}\times A^{2}+4.092\times B^{2}+0.1218\times C^{2}+8.604\times D^{2} \end{array}$$

(7) $$\begin{array}{l} Kerf\;taper\left(Degree\right)=-542.946+0.107\times A+45.884\times B+12.156\times C\\ +25.781\times D-0.0151\times A\times B-1.018\times 10^{-3}\times A\times C+0.204\times B\times C\\ +3.968\times B\times D-0.776\times C\times D-4.335\times B^{2}-0.0843\times C^{2} \end{array}$$

(8) $$\begin{array}{l} HAZ\left(mm\right)=82.091+0.0341\times A+5.167\times B-4.350\times C-10.578\times D\\ -1.98\times 10^{-3}\times A\times B+6.337\times 10^{-4}\times A\times C-2.287\times 10^{-3}\times A\times D\\ -0.795\times B\times D+0.274\times C\times D-1.128\times 10^{-5}\times A^{2}+0.022\times C^{2}+0.819\times D^{2} \end{array}$$

**Figure 4:**
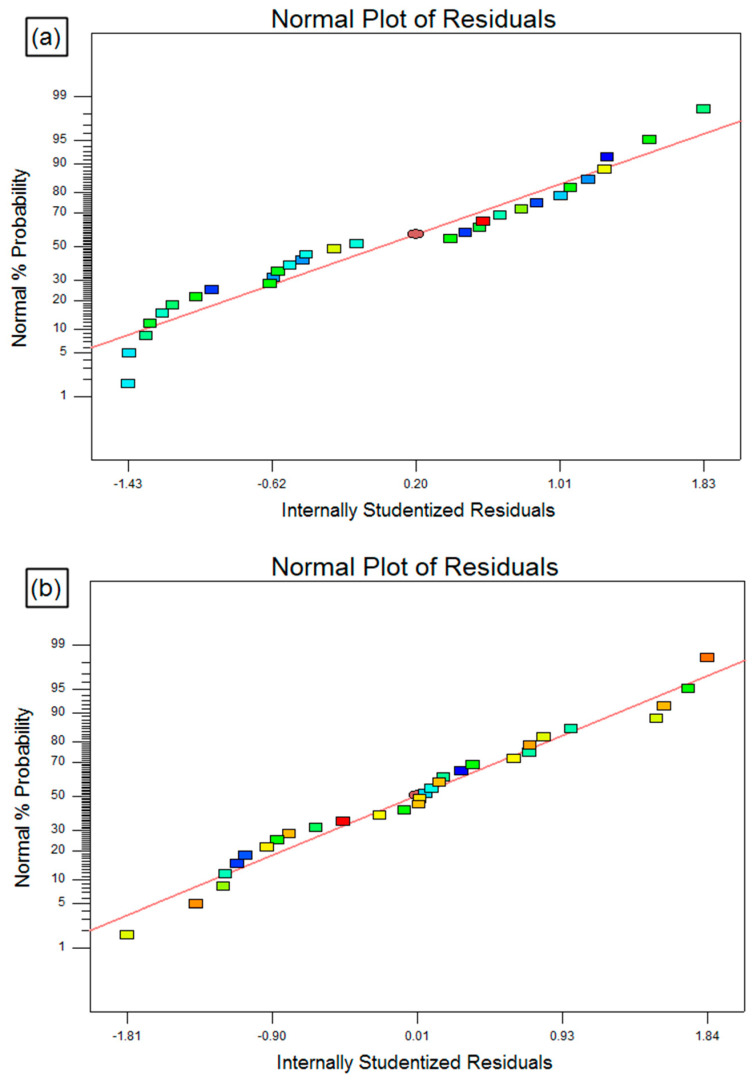

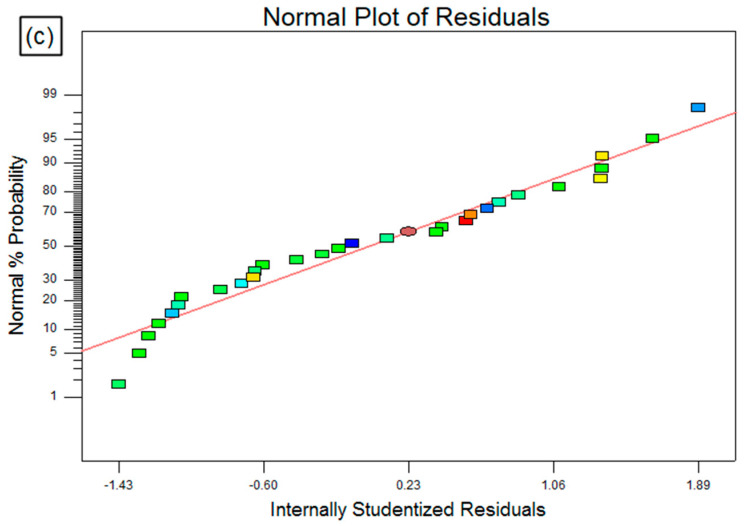
Normal probability residual plots for (**a**) material removal rate (MRR), (**b**) kerf taper (KT), and (**c**) heat affected zone (HAZ).

Moreover, the normal probability plots shown in Figure 4a–c indicated that the residuals are dispersed normally, which implies that the proposed models are legitimately accurate and acceptable. Hence, the established second-order mathematical equations are realistically satisfactory in place of the PAC process.

### 4.2. Fuzzy Modeling of PAC Process Parameters

**Figure 5:**
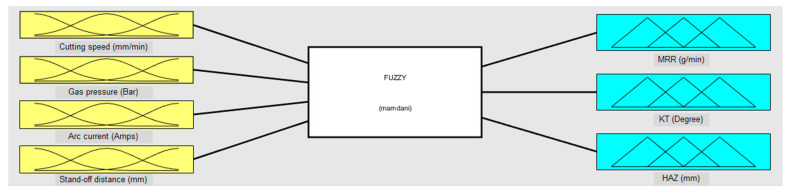
Model representation of PAC input variables and output responses using the Mamdani fuzzy logic approach.

**Figure 6:**
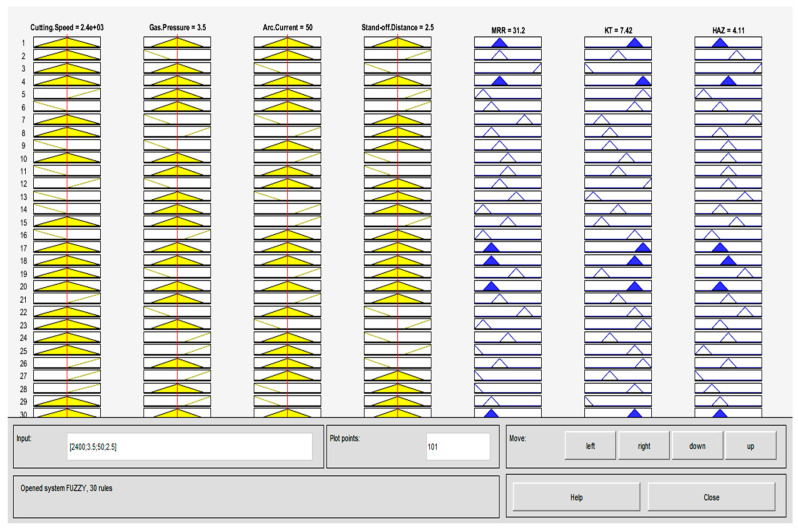
Fuzzy rule viewer with a triangular membership function.

With a due consideration on four input PAC process variables and three output responses (Figure 5), thirty fuzzy rules are formulated using the Mamdani approach to build an inter-relationship between them which is shown in Figure 6. Here, the triangular shape is used as a membership function because of its advantage of simplicity. Thus, for the four input parameters and their three-membership values, the number of fuzzy rules formed are 30. From the fuzzy rule viewer, the rows are considered to be the developed rules and columns signify input and output variables and also the MF values are represented as the height of triangles. Each triangle location indicates the input and output response and the height of darkened region of the triangle corresponds to the fuzzy membership value. After performing the simulation, defuzzified values for MRR of 31.2 g min^−1^, KT of 7.42°, and HAZ of 4.11 mm are obtained for the given input process variables: Cutting speed = 2400 mm min^−1^, gas pressure = 3.5 bar, arc current = 50 A, and stand-off distance = 2.5 mm in the 25th experimental run as presented in Table 2.

**Figure 7:**
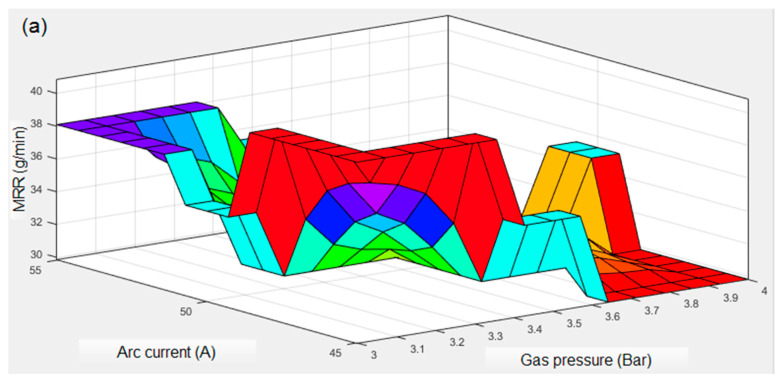

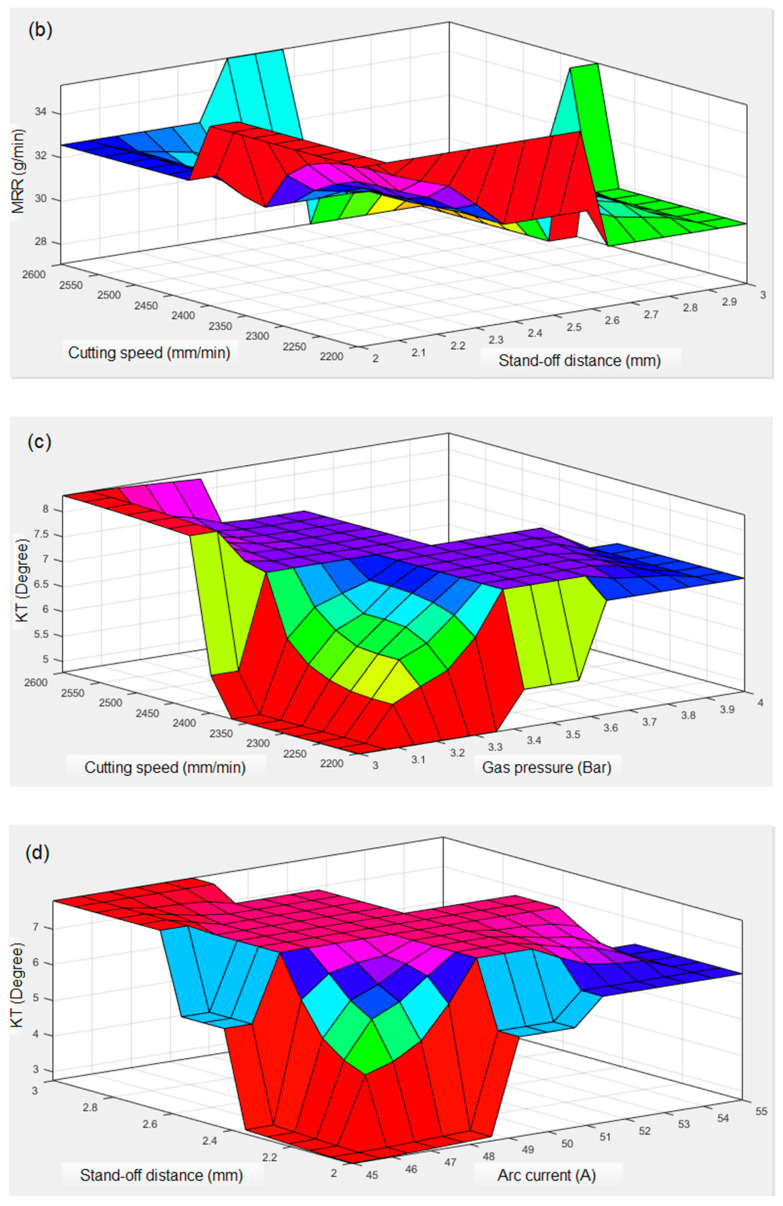

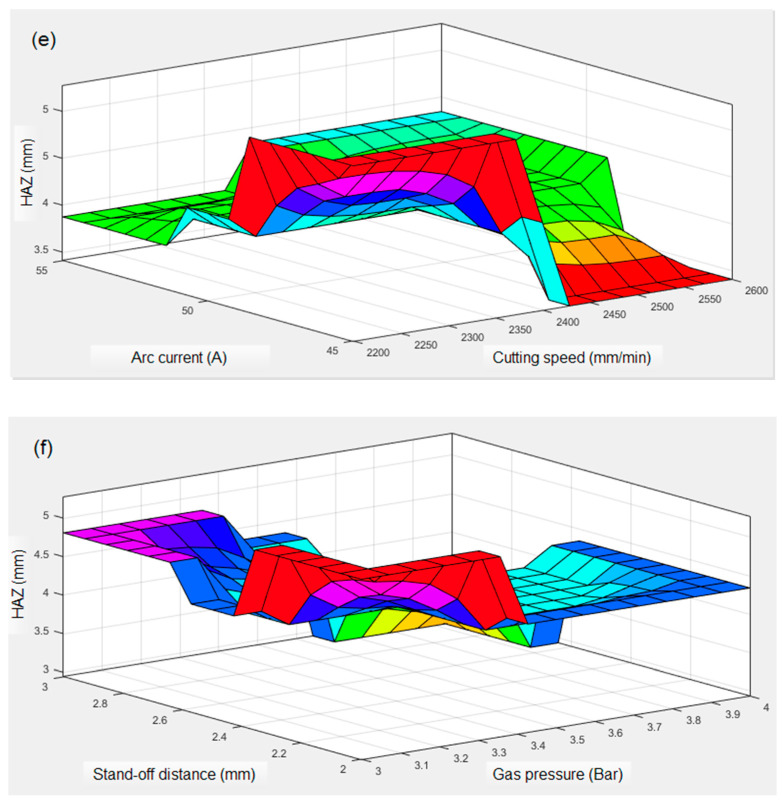
Three-dimensional (3D) fuzzy surface plots on various responses: (**a**,**b**) for MRR, (**c**,**d**) for KT, and (**e**,**f**) for HAZ.

Three-dimensional fuzzy plots shown in Figure 7a–f indicate the impact of PAC governing parameters on MRR, KT, and HAZ. The influence of arc current and gas pressure on MRR shown in Figure 7a describes that MRR decreases with a rise in gas pressure (3.5 to 4 bar) and it increases with a surge in arc current from 45 to 55 A. It is due to the fact that a high concentration of plasma energy is transferred to the workpiece at higher arc current which leads to quick melting and the vapourization of metal resulted in higher MRR.

**Figure 8:**
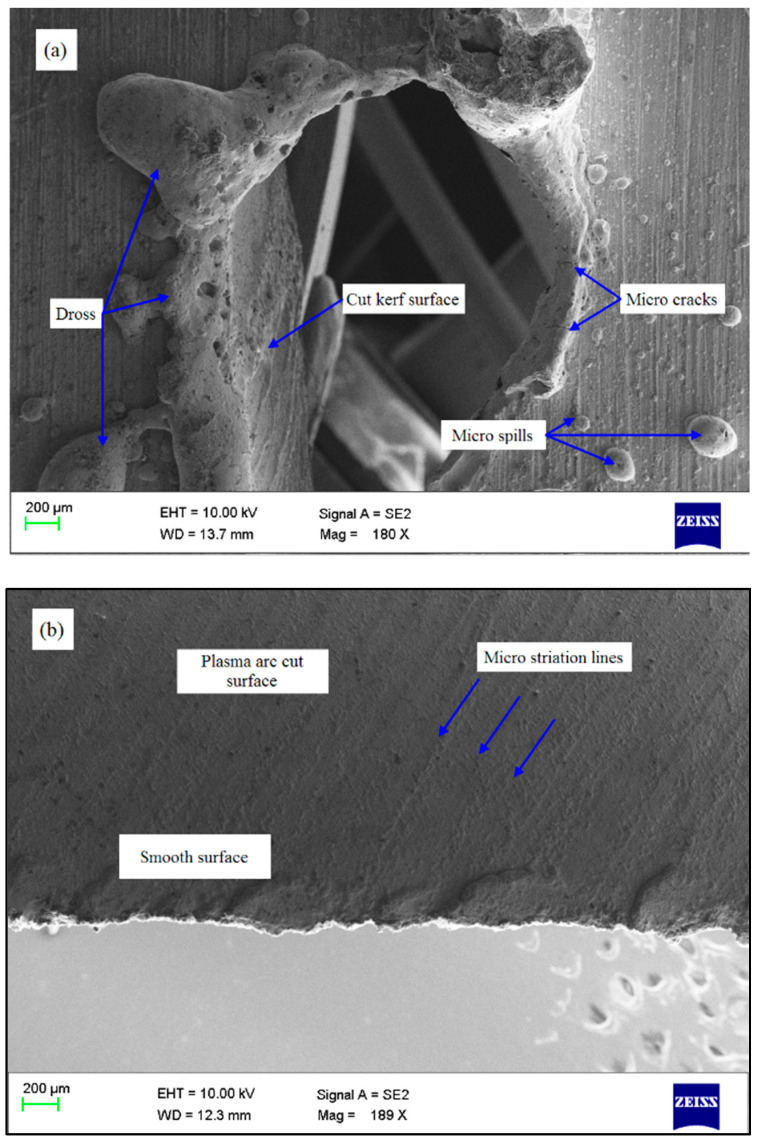

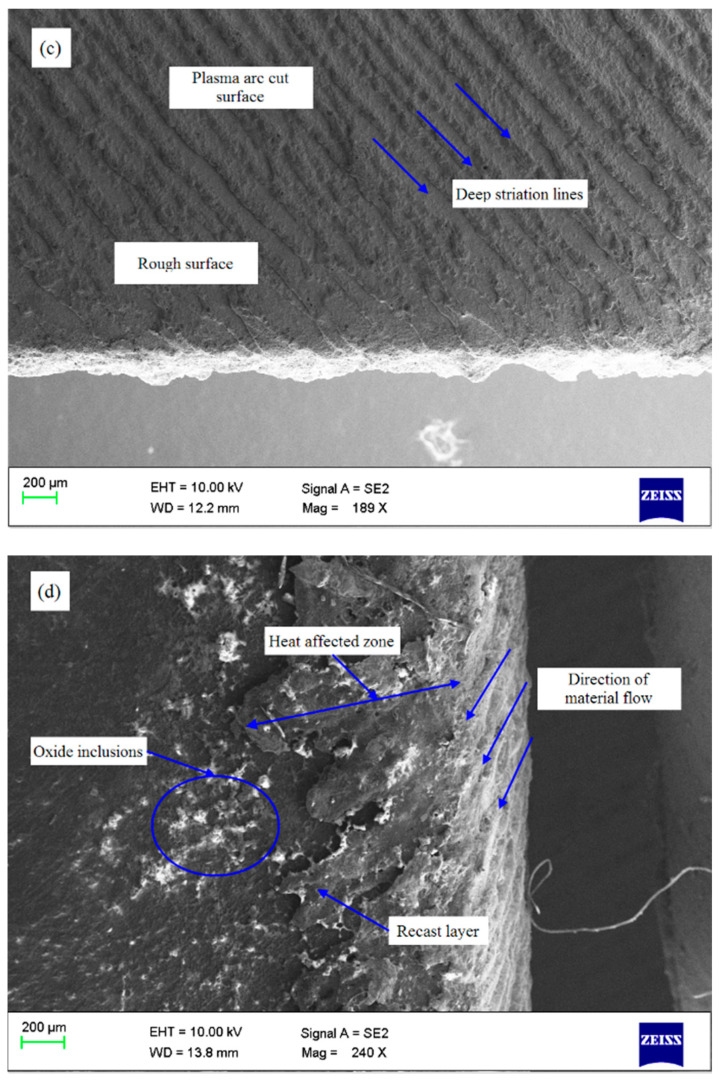
Morphological examination of plasma cut surfaces, (**a**) at higher cutting speed of 2600 mm min^−1^, (**b**) at lower cutting speed (2200 mm min^−1^) and gas pressure (3 bar), (**c**,**d**) at higher stand-off distance of 3 mm.

Figure 7b exhibits the variation in MRR as a function of change in cutting speed and stand-off distance. It is evident that the maximized MRR is obtained at the combination of lower cutting speed (2200 mm min^−1^) with a lower stand-off distance (2 mm). Increasing of the cutting speed and stand-off distance beyond 2400 mm min^−1^ and 2.5 mm, respectively reduces the removal of material. At higher cutting speed and stand-off distance, heat energy ejected from the plasma is not sufficiently transferred to the interaction zone that ensures a low material interaction time and hence MRR decreases. Microstructural observation (Figure 8a) of the top cut surface reveals that the enlarged keyhole and higher dross formation is due to the reduction in kinetic energy of plasma at obtrusion when the cutting speed increases (2600 mm min^−1^). During lower cutting speed, perpendicular draglines are formed on the workpiece due to a sudden increase in the arc current. Dross formation occurs at the bottom edge of the cut surface during high speed cutting and it varies according to the speed. At low speed, the kerf gets wider and becomes hardened where in the jet it was unable to flash out the smelted material. However, at high cutting speed, the unstable arc is formed without an increase in the current [17].

The impact of cutting speed and gas pressure on KT depicted in Figure 7c signifies that, KT becomes wider with an increase of these parameters, whereas a lower KT is observed at low cutting speed (2200 mm min^−1^) and gas pressure (3 bar). Above this cutting speed, the torch travels along the workpiece at a faster rate to maintain the plasma arc stability. However, it may not be able to remain perpendicular to the cutting edge causing a wider kerf [8,12]. However, the cut surface is more consistent and the accurate kerf is obtained during low cutting speed and gas pressure. From the micrograph of kerf side surface (Figure 8b), a reduced striation pattern with fewer molten pool adherence and enhanced surface finish is observed [10].

Figure 7d portrays the discrepancy of KT as a function of the stand-off distance and arc current. It can be noted that a minimal KT is attained through upholding lower stand-off distance and arc current, whereas an increase in these parameters caused the widening of KT. At high stand-off distance and arc current, the uncertainty of plasma arc occurs leading to a lack of expelled energy from the plasma nozzle. Consequently, an indiscriminate melting and eradication of molten material transpires, moreover the plasma arc cannot extend the bottom of the sheet metal with the necessary energy density which results in the increased kerf width [31,32]. Examining the side cut surface conditions through the SEM analysis (Figure 8c), deep striation lines are observed due to the flow of re-solidified molten metal at a higher stand-off distance. An increase in the stand-off distance resulted in the long curve of plasma arc and drag-lines, and the surface waves are formed over the workpiece surface. The same trending has been found by Salonitis and Vatousianos [10].

The effect of arc current and cutting speed on HAZ is shown in Figure 7e revealing that, HAZ decreases with an increase in the arc current and cutting speed. A minimal HAZ is obtained during the combination of higher cutting speed with lower arc current. However, at greater cutting speed, heat that inflows into the workpiece is reduced which decreases the size of HAZ [10]. Moreover, an increase in the arc current leads to the penetration of more heat energy into the workpiece causing a larger HAZ [8].

The cumulative impact of stand-off distance and gas pressure on HAZ is presented in Figure 7f demonstrating that HAZ decreases with an increase in gas pressure from 3 to 3.5 bar and a further increase in gas pressure has not affected HAZ. However, an increase in the stand-off distance from 2 to 2.7 mm resulted in a decrease in HAZ and a further increase in the stand-off distance improved the HAZ. It might be due to the fact that expansion of plasma arc before impingement of the workpiece during a higher stand-off distance leads to higher HAZ. Subsequently, the morphology of the top cut surface at high stand-off distance depicted in Figure 8d denotes an improved heat affected region with oxide inclusions owing to the increased jet diameter. Therefore, it is expected to reduce the stand-off distance and increase the gas pressure to increase the cut quality [15] by means of decreasing HAZ.

### 4.3. Assessment of Fuzzy and Response Surface Models

To investigate the prediction accuracy of the developed fuzzy logic and RSM approach, the results predicted by FL and RSM are validated using statistical methods via investigating the determination of coefficients (R^2^) and root-mean-square error (RMSE), using the following relations:

(9) $$R^{2}=1-\frac{\sum_{i=1}^{n}{\left(S_{Exp}-S_{\mathrm{Pr}}\right)}^{2}}{\sum_{i=1}^{n}{\left(S_{Exp}-\bar{S_{\mathrm{Pr}}}\right)}^{2}}$$

(10) $$RMSE={\left[\frac{1}{M}\sum_{i=1}^{m}\left(S_{Exp}-S_{\mathrm{Pr}}\right)\right]}^{\frac{1}{2}}$$

where $S_{Exp}$ indicates experimental measured value, $S_{\mathrm{Pr}}$ specifies fuzzy or RSM predicted value, and M is the number of experiments performed. The estimated outright fraction of variance and RMSE values for selected responses using fuzzy and RSM models are presented in Table 4. It is evident that the assessed RMSE and R^2^ values using the fuzzy logic approach for MRR, KT, and HAZ are minimal in comparison to the anticipated RSM values. Therefore, the established fuzzy approach is efficiently used to predict multi-response characteristics through varying PAC process parameters without performing experimental studies within the selected bounds of parameters.

**Figure 9:**
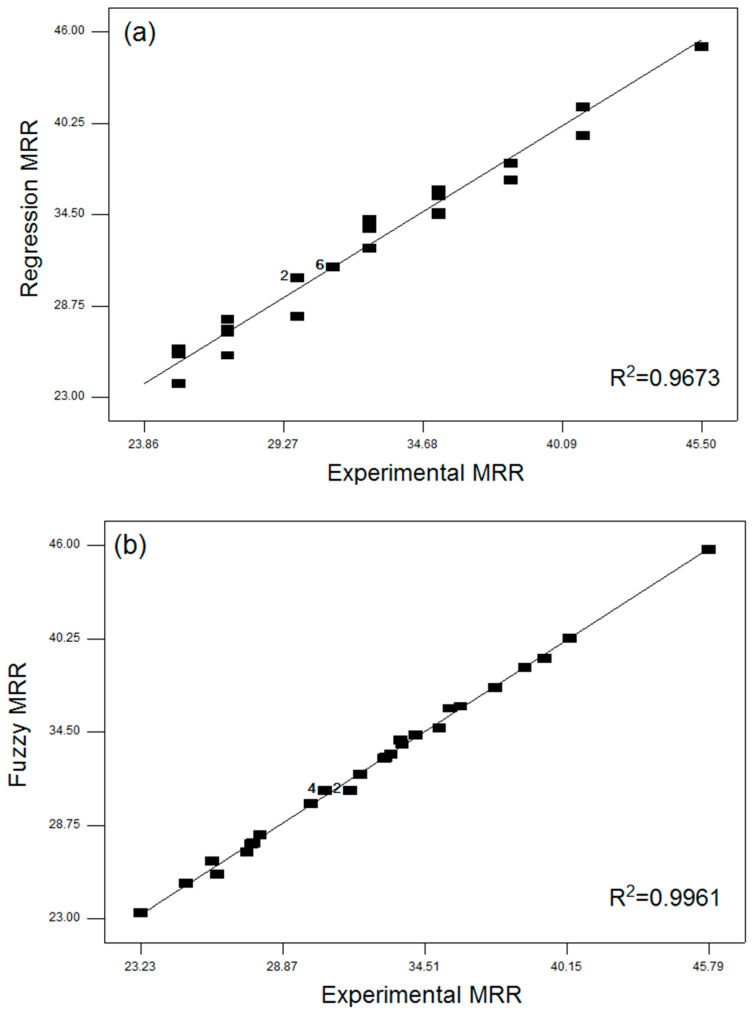

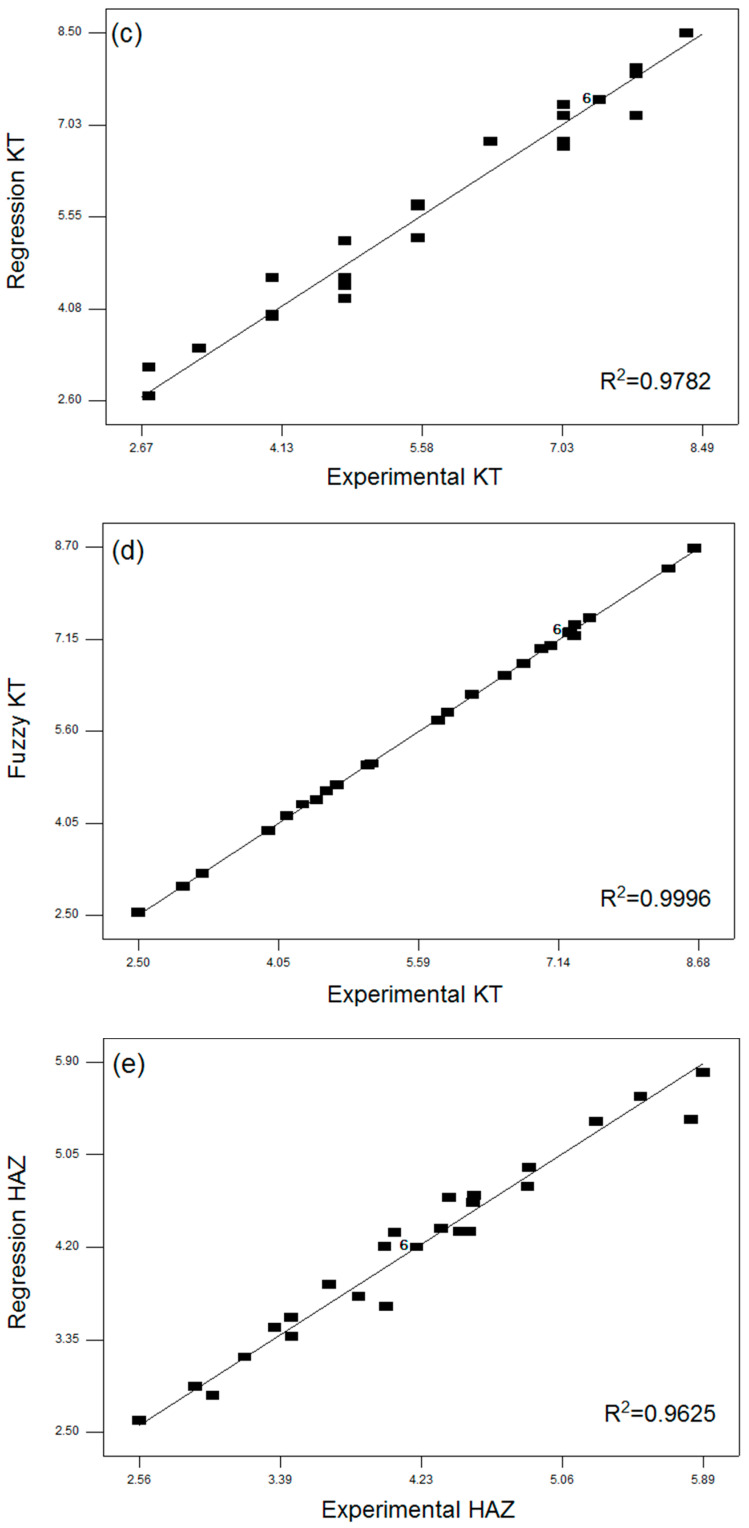

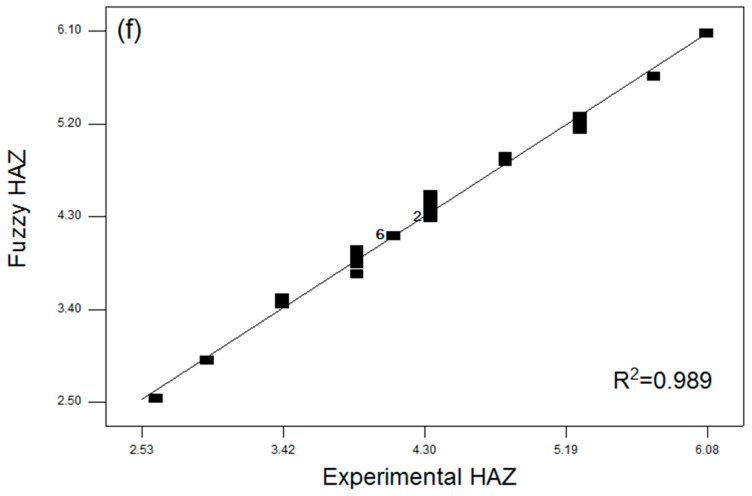
(**a**–**f**). Comparison of regression and fuzzy models with experiments on MRR, KT, and HAZ.

The performance validation of developed fuzzy and response surface models with experimental results for MRR, KT, and HAZ are graphically presented in Figure 9a–f. Figure 9a,b indicates the comparison between normal probability plots of regression and fuzzy predicted values for MRR, Figure 9c,d indicates the comparison between normal probability plots of regression and fuzzy predicted values for KT and Figure 9e,f indicates the comparison between normal probability plots of regression and fuzzy predicted values for HAZ, respectively. From the figures, it is found that the fuzzy and RSM predicted values are closely scattered on both sides on a 45° inclined line that endorses the unified capability of the established methods. In addition, the fuzzy algorithm achieved a better and accurate prediction than the RSM with a high R^2^ value.

Moreover, in order to evaluate the accuracy and efficiency of developed models in terms of predicting MRR, KT, and HAZ, the average prediction error is estimated and calculated using the following relation:

(11) $$Avg.\;prediction\;error\left(\lambda \right)=\frac{S_{\mathrm{Pr}}-S_{Exp}}{S_{Exp}}\times 100$$

**Figure 10:**
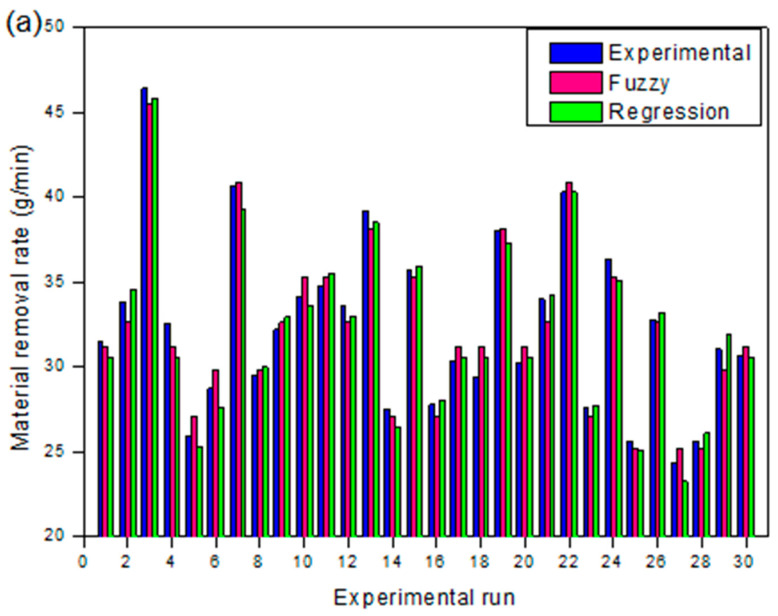

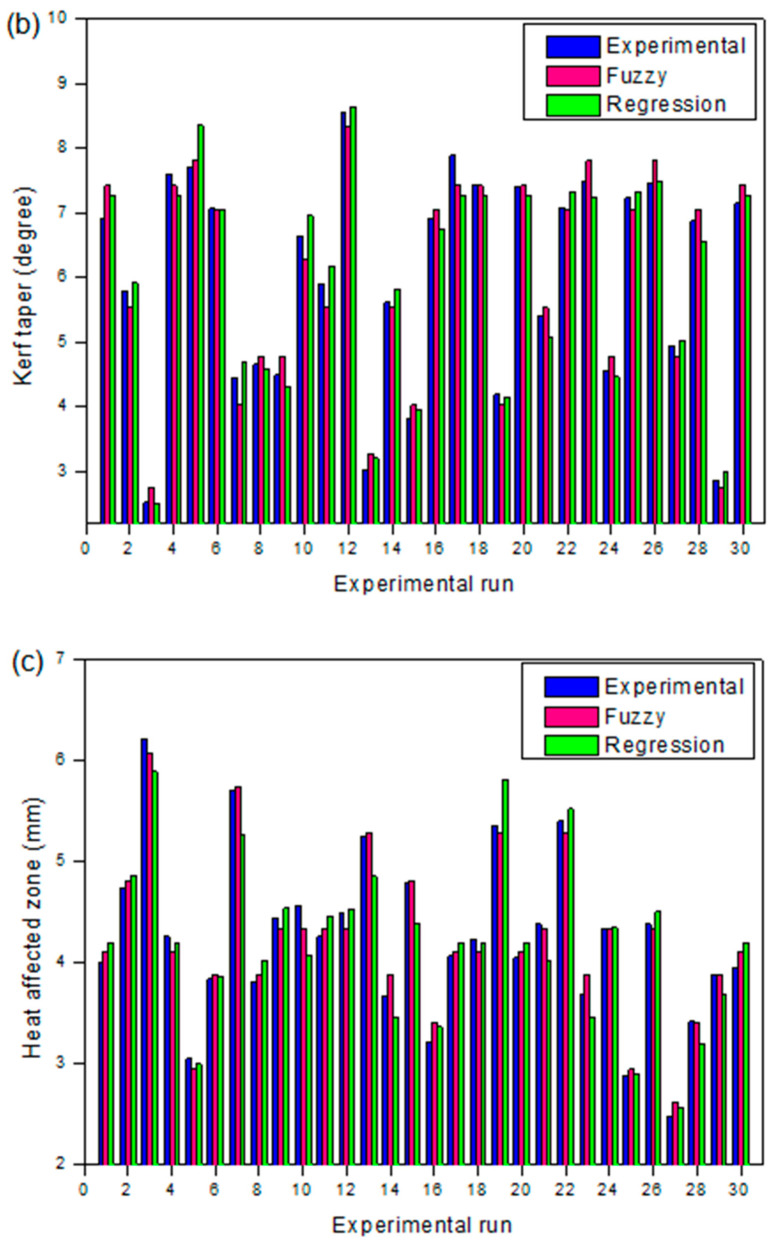
Correlation between fuzzy and regression predicted response values with experimental results for (**a**) MRR, (**b**) KT, and (**c**) HAZ.

The average error observed (Figure 10a–c) between the fuzzy and experimental values are about 0.04% for MRR, 0.48% for KT, and 0.46 for HAZ and between the RSM predicted and experimental results are about 0.51%, 0.51%, and 0.68% for MRR, KT, and HAZ, respectively.

Therefore, it is observed that the predicted values by the fuzzy approach was more accurate than RSM. Due to the low order non-linear behaviour of RSM having a trivial factors region, it is cumbersome to fit the data over an asymmetrical investigational dominion. Hence, the prediction ability of the RSM technique declines due to the complexity in the PAC process and the fuzzy approach exhibits a more accurate prediction that even many experiments are considered for the development of the fuzzy based model. Though, generation of a fuzzy rule requires experimentation and experience which lead to high computational time than a response surface model.

### 4.4. Sensitivity Analysis

The sensitivity analysis is used to examine the response of PAC variables qualitatively and quantitatively and also provide ranking through their order of performance. It has significance in validating the model where efforts are attempted to associate the deliberated output to the distinguished data [33]. The objective function for this analysis is derived from the fractional derivative of the function under consideration to the equivalent process variables [34].

The current study aims to investigate the affinity of MRR, KT, and HAZ with reference to diverse PAC process variables such as cutting speed, gas pressure, arc current, and stand-off distance. The facts of sensitivity can be inferred by means of a mathematical representation of derivatives. The optimistic sensitivity of certain process variables for an objective indicates an augmentation in the objective due to a variation in design parameters, whereas adverse sensitivity indicates the contrary.

Through partially differentiating the equations (Equations (6)–(8)) with respect to four accounted PAC process variables, sensitivity relationships are established (Equations (12)–(23)). They are further utilized to predict the variation in response with respect to each process variable:

(12) $$\frac{\partial S_{MRR}}{\partial A}=0.0623-0.01205B+0.005028C-0.000116A$$

(13) $$\frac{\partial S_{MRR}}{\partial B}=3.757-0.01205A-4.327D+8.184B$$

(14) $$\frac{\partial S_{MRR}}{\partial C}=-29.547+0.005028A+2.041D+0.2436C$$

(15) $$\frac{\partial S_{MRR}}{\partial D}=-137.812-4.327B+2.041C+17.208D$$

(16) $$\frac{\partial S_{KT}}{\partial A}=0.107-0.0151B-0.00108C$$

(17) $$\frac{\partial S_{KT}}{\partial B}=45.884-0.0151A+0.204C+3.968D-8.67B$$

(18) $$\frac{\partial S_{KT}}{\partial C}=12.156-0.001018A+0.204B-0.776D-0.1686C$$

(19) $$\frac{\partial S_{KT}}{\partial D}=25.781+3.968B-0.776C$$

(20) $$\frac{\partial S_{HAZ}}{\partial A}=0.0341-0.00198B+0.00064C-0.0023D-0.00023A$$

(21) $$\frac{\partial S_{HAZ}}{\partial B}=5.167-0.00198A-0.795D$$

(22) $$\frac{\partial S_{HAZ}}{\partial C}=-4.35+0.00064A+0.274D+0.044C$$

(23) $$\frac{\partial S_{HAZ}}{\partial D}=-10.578-0.0023A-0.795B+0.274C+1.638D$$

**Figure 11:**
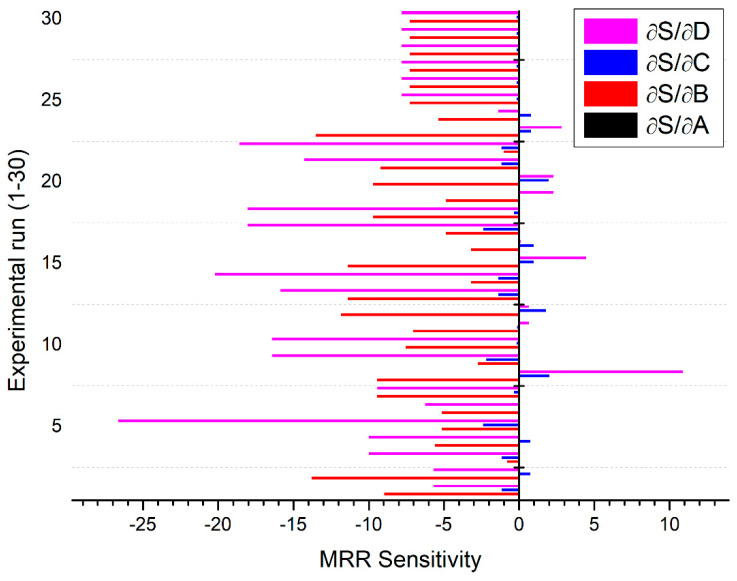
Sensitivity of PAC variables on MRR.

**Figure 12:**
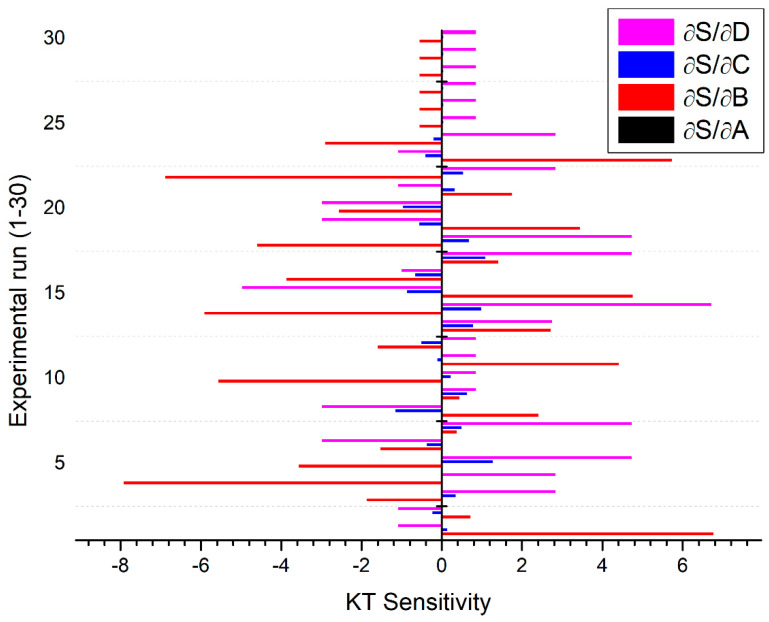
Sensitivity of PAC variables on KT.

**Figure 13:**
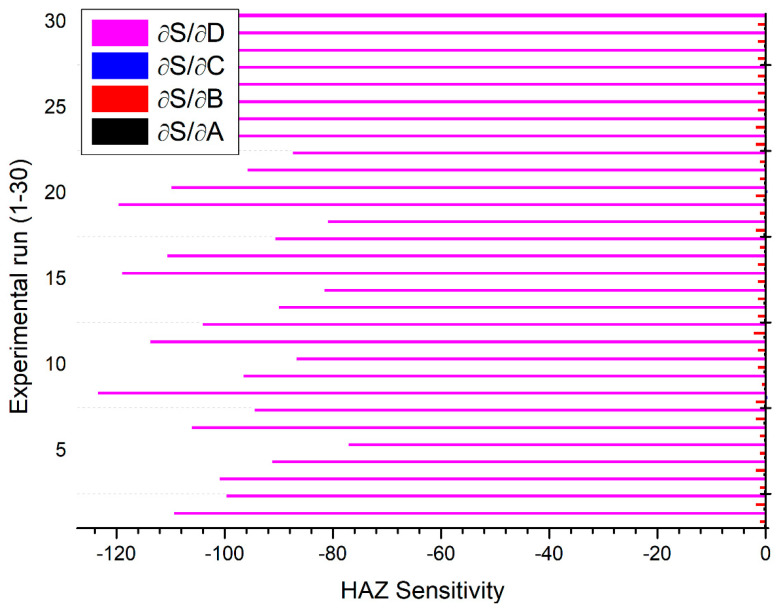
Sensitivity of PAC variables on HAZ.

The sensitivity analysis results are presented in Figure 11, Figure 12 and Figure 13 by solid bars with respect to different settings of plasma arc cutting as premeditated using the design matrix shown in Table 2. It is observed from Figure 11 that, MRR is found to be more sensitive to the stand-off distance in comparison to other process parameters. However, KT is highly sensitive to the gas pressure followed by variation in the stand-off distance (Figure 12). In particular, the gas pressure is more sensitive in the negative direction whereas the stand-off distance is sensitive in the positive direction. It signifies that, KT increases with an increase in the stand-off distance and decreases with an increase in the gas pressure. Figure 13 depicted that HAZ is highly sensitive to the variation in the stand-off distance in a negative sense than other parameters. The sensitivity analysis results reported in Figure 11, Figure 12 and Figure 13 exposed that both stand-off distance and gas pressure partake an inordinate impact on quality and performance characteristics, which can be in tune to regulate the superiority of PAC end use components.

## 5. Conclusions

Investigations are accomplished to examine the effect of PAC system variables on MRR, KT, and HAZ through developing a regression and fuzzy logic expert system. The PAC experiments were planned and conducted using the BBD-RSM approach. Based on mathematical and stochastic modeling and also experimental analysis, the following conclusions are drawn:

- The ANOVA table reveals that the developed quadratic models for MRR, KT, and HAZ are found to be adequate with an experimental result within 95% of assurance level to envisage the responses precisely within the boundaries of considered PAC variables.
- Experimental results showed that MRR improves with an increase in the arc current and stand-off distance, whereas it significantly decreases with an increase in the cutting speed and gas pressure. KT is found to be minimal when all the selected parameters are kept at a lower level. The combination of higher cutting speed and lower arc current with intermediate values of gas pressure and stand-off distance produced lower HAZ.
- Morphological examination of cut surfaces reveals that, the presence of striation lines, dross formation, and micro cracks significantly affected the surface quality.
- The average prediction error between the fuzzy and experimental values are 0.04% for MRR, 0.48% for KT, and 0.46 for HAZ, whereas the average error observed between regression models and experimental results are 0.51%, 0.51%, and 0.68% for MRR, KT, and HAZ, respectively. It is evident that the fuzzy logic expert system is found to be superior in predicting the responses in PAC of the Monel 400 alloy.
- The sensitivity analysis results suggested that the stand-off distance (+12 to −25) is the most sensitive parameter to MRR. The gas pressure (+7 to −8) and stand-off distance (+7 to −5) are more sensitive to KT, whereas the stand-off distance (−123) is a highly sensitive parameter to HAZ.

## Figures and Tables

**Table 1:** Plasma arc cutting variables and their levels.

S. No.	Process Variables	Levels			Unit
		Low	Medium	High	
1	Cutting speed (A)	2200	2400	2600	mm min^−1^
2	Gas pressure (B)	3	3.5	4	Bar
3	Arc current (C)	45	50	55	A
4	Stand-off distance (D)	2	2.5	3	mm

**Table 2:** Experimental design and measured response values.

Run	Input Parameters				Responses		
	Cutting Speed (mm min^−1^)	Gas Pressure (Bar)	Arc Current (A)	Stand-off Distance (mm)	MRR (g min^−1^)	KT (Degree)	HAZ (mm)
1	2200	3	50	2.5	32.183	4.491	4.44
2	2600	3	50	2.5	33.57	8.557	4.5
3	2200	4	50	2.5	27.753	6.912	3.21
4	2600	4	50	2.5	24.319	4.935	2.475
5	2400	3.5	45	2	46.371	2.52	6.21
6	2400	3.5	55	2	34.107	6.632	4.56
7	2400	3.5	45	3	27.572	7.472	3.69
8	2400	3.5	55	3	35.718	3.815	4.785
9	2200	3.5	50	2	34.779	5.897	4.26
10	2600	3.5	50	2	32.764	7.455	4.38
11	2200	3.5	50	3	28.736	7.07	3.84
12	2600	3.5	50	3	25.899	7.707	3.045
13	2400	3	45	2.5	40.611	4.447	5.7
14	2400	4	45	2.5	31.004	2.87	3.885
15	2400	3	55	2.5	38.015	4.185	5.355
16	2400	4	55	2.5	29.467	4.655	3.81
17	2200	3.5	45	2.5	39.209	3.027	5.25
18	2600	3.5	45	2.5	25.602	6.874	3.42
19	2200	3.5	55	2.5	27.482	5.615	3.675
20	2600	3.5	55	2.5	33.989	5.39	4.38
21	2400	3	50	2	40.284	7.083	5.4
22	2400	4	50	2	36.345	4.55	4.335
23	2400	3	50	3	33.84	5.792	4.74
24	2400	4	50	3	25.574	7.227	2.88
25	2400	3.5	50	2.5	31.423	6.907	4.005
26	2400	3.5	50	2.5	32.554	7.591	4.26
27	2400	3.5	50	2.5	30.629	7.145	3.945
28	2400	3.5	50	2.5	30.347	7.892	4.065
29	2400	3.5	50	2.5	30.257	7.402	4.05
30	2400	3.5	50	2.5	29.364	7.437	4.23

**Table 3:** Analysis of variance and model validation.

Source	Sum of Square	DOF	Mean Square	F	Prob. > F	R2	Adj. R2	Adeq. Precision
MRR								
Model	742.06	14	53	45.96	<0.0001	0.9772	0.956	29.68
Total	759.36	29						
Residual	17.3	15	1.15					
Lack of fit	11.25	10	1.13	0.93	0.5706			
Pure error	6.05	5	1.21					
KT								
Model	79.64	14	5.69	65.39	<0.0001	0.9839	0.9688	29.625
Total	80.95	29						
Residual	1.3	15	0.087					
Lack of fit	0.72	10	0.072	0.61	0.7637			
Pure error	0.59	5	0.12					
HAZ								
Model	19.66	14	1.4	109.99	<0.0001	0.9904	0.9813	46.155
Total	19.85	29						
Residual	0.19	15	0.013					
Lack of fit	0.11	10	0.011	0.71	0.6972			
Pure error	0.079	5	0.016					

**Table 4:** Comparison of fuzzy and RSM responses.

Models	MRR		KT		HAZ	
	R^2^	RMSE	R^2^	RMSE	R^2^	RMSE
Fuzzy	0.9961	2.51	0.9996	3.9	0.989	2.95
RSM	0.9673	2.8	0.9782	4.66	0.9625	5.24
